# Bacterial cellulose: A highly versatile nanomaterial

**DOI:** 10.1111/1751-7915.14243

**Published:** 2023-03-09

**Authors:** Sophie‐Marie Martirani‐VonAbercron, Daniel Pacheco‐Sánchez

**Affiliations:** ^1^ Estación Experimental del Zaidín, Department of Environmental Protection Consejo Superior de Investigaciones Científicas Granada Spain

Bacterial cellulose (BC) is a natural and renewable nanomaterial characterized by a unique three‐dimensional structure that endows it with excellent mechanical properties and high water‐holding capability. BC is a very pure, crystalline and biocompatible polymer. Moreover, BC can be modified to improve its innate features and to provide it with tailored properties or new functionalities which could be applicable in many areas. Due to these outstanding proprieties, BC is receiving a great deal of attention in both academic and industrial fields. Here, we highlight various recent studies and reviews that discuss the molecular aspects of BC biosynthesis and its regulation and summarize the most common commercial applications of BC in the biomedical and textile areas. There is great expectation that BC will be developed to its full potential, and become a modifiable material of choice depending on the demands of the different areas of use.

Cellulose is the most abundant biopolymer on Earth and while it is the predominant building constituent of plants, cellulose biosynthesis is not only limited to plants. Its production has been reported in a vast range of bacteria, protists, fungi, algae, and even some giant viruses (*Pandoravirus* genus) that incorporate cellulose into their tegument (Brahim Belhaouari et al., 2019). Among bacteria, there are many genera which are BC producers that inhabit diverse ecological niches including fruit colonizers (*Komagataeibacter*), soil bacteria (*Burkholderia* and *Pseudomonas*), plant pathogens (*Agrobacterium* and *Dickeya*), plant symbionts (*Rhizobium* and *Azotobacter*), animal pathogens (*Escherichia*, *Enterobacter*, *Salmonella* and *Alcaligenes*) and highly metabolically versatile soil bacteria (*Starkeya*; Figure 1; Augimeri et al., 2015; Manan et al., 2022; Marín et al., 2019; Römling & Galperin, 2015). In recent years, the interest in BC biosynthesis has increased, essentially based on intensive studies on *Komagataeibacter* as a model organism, due to its high cellulose production yield. This has helped us to understand the BC biosynthesis mechanisms and to design new possible applications and improvements for this useful biopolymer. The global BC market size is estimated to reach USD 777 million by 2027, with a growth rate of about 38% during 2019–2027 (Manan et al., 2022). The rapid growth of the global BC market encourages the development of new technologies that could enhance production processes and meet the BC market demand.

**FIGURE 1:**
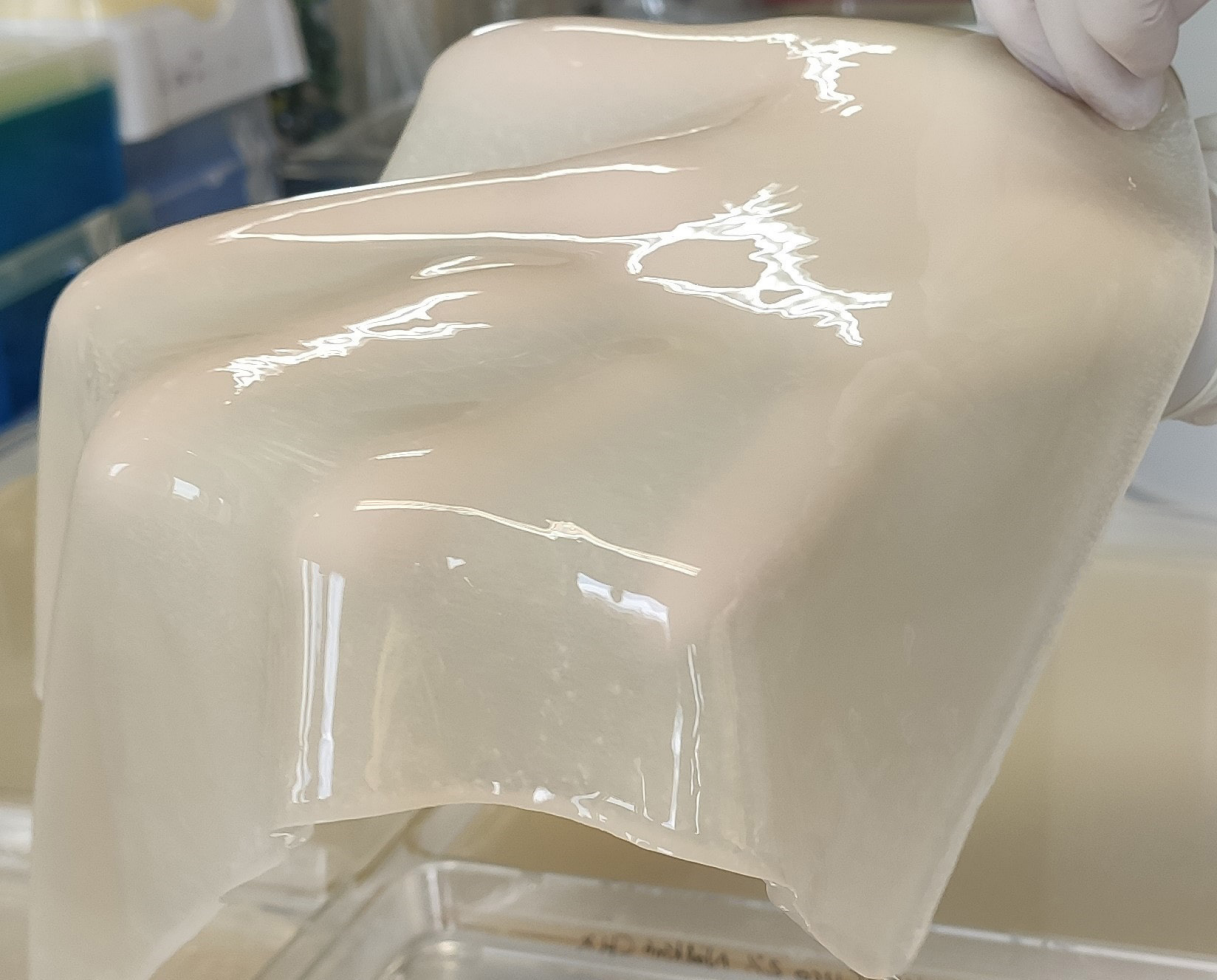
Bacterial cellulose production by *Starkeya* sp.

The chemical structure of BC consists of linear chains of β‐D‐glucopyranose units joined via β‐1,4‐glycosidic linkages. BC biosynthesis is a three‐step process: (i) polymerization of glucose units and production of β‐1,4‐glucan chains, (ii) extracellular transport and (iii) crystallization of cellulose (Manan et al., 2022). The synthesis process is carried out by membrane‐integrated cellulose synthase (CS) complexes, encoded by *bcsABCD* operons. CSs use UDP‐glucose as a substrate, the common cellulose precursor in most organisms. UDP‐glucose can be obtained through different interconnected metabolic pathways and using different carbon sources, such as glucose, fructose, hexose, sucrose, glycerol, dihydroxyacetone, pyruvate and dicarboxylic acids, which is advantageous from a biotechnological point of view (Jacek et al., 2019). The physiological state of the cell can determine the flux from these pathways to UDP‐glucose, and thus, the amount of BC to be synthesized. As an example, the interdependence between the catabolic pathways of ethanol, glucose and fructose is proposed in the metabolic model of *Komagataeibacter entanii*. The authors observed that all four metabolic products (biomass, acetic acid, acetan and cellulose) can be produced by any of the carbon sources, although with different energy balances (Velasco‐Bedrán & López‐Isunza, 2007). Hence, understanding the metabolism of the BC‐producing strains offers a tool to apply metabolic engineering for the construction of improved strains.

Our knowledge on the molecular structure of BC synthase machinery has increased significantly thanks to molecular techniques, the characterization of proteins involved in the biosynthesis of BC and the construction of genetically modified strains. Genetic and molecular studies indicate the existence of three major types of cellulose secretion systems, the best characterized being the type I system found among certain α‐, β‐ and γ‐Proteobacteria, including *Komagataeibacter xylinus* (Römling & Galperin, 2015). In most bacteria, the catalytic core of CS is the BcsAB tandem anchored in the inner cell membrane. BcsA presents a transmembrane region and two large cytoplasmic domains, a glycosyltransferase domain and the PilZ regulatory domain that binds c‐di‐GMP. BcsB is a periplasmic subunit anchored to the inner membrane by a single transmembrane helix that carries out the transport of the newly synthesized β‐1,4‐glucan chain from the cytoplasm through the periplasmic space (Abidi et al., 2022; McNamara et al., 2015). In many cellulose‐secreting Gram‐negative bacteria, *bcs* operons also encode the outer membrane exporter protein BcsC and the periplasmic homologues of the cellulase, termed BcsZ or CMCax. The BcsC subunit is probably involved in the extrusion of cellulose through the outer membrane and CMCax possesses an endo β‐1,4‐glucanase activity and plays a role in regulating cellulose production (Acheson et al., 2019). Type I cellulose secretion systems also include the periplasmic BcsD subunit that is proposed to contribute to the regular packing of glucan chains and the formation of crystalline regions of the cellulose chain (Hu et al., 2010; Römling & Galperin, 2015). The *bcs* operon in *Komagataeibacter* genus contains flanking genes such as *ccpAx* and *bglAx*. The function of CcpAx is not clear, although it has been suggested that it interacts with the BcsD subunit and participates in the activation of CS activity (McManus et al., 2016). BglAx is a non‐essential enzyme with β‐glucosidase activity; it probably plays a similar role to that of CMCax, as a cellulose‐digesting enzyme controlling the quality of the secreted polysaccharide (Abidi et al., 2022; Jacek et al., 2019).

It has long been known that BC biosynthesis is regulated at both the transcriptional and post‐translational levels. A main player in the regulatory system is the second messenger c‐di‐GMP, required as an allosteric activator of BcsA through binding to its cytoplasmic PilZ domain. Once c‐di‐GMP binds to the PilZ domain, the BcsA active site becomes accessible for its UDP‐glucose substrate. In many BC‐producing bacteria, c‐di‐GMP cellular levels are modulated by the antagonistic action of two enzymes, diguanylate cyclase which synthesizes c‐di‐GMP and phosphodiesterase which hydrolyses c‐di‐GMP (Morgan et al., 2014).

Several researchers have attempted to improve BC production yield. In general, growth conditions greatly affect the overall BC production, and process optimization is feasible. However, it is still not possible to fully control the biosynthesis and properties of the cellulose produced by *Komagataeibacter* strains. The genetic instability of BC‐producing bacterial cells is an important hurdle that prevents the achievement of high cellulose production rates. The appearance of spontaneous Cel – mutants that do not produce cellulose during shaken cultivation makes it even more complicated (Jacek et al., 2019; Manan et al., 2022). Nevertheless, in recent years, numerous complete genome sequences of BC‐producing bacteria have been obtained, providing background information for the genetic engineering required for the precise control of BC biosynthesis (Manan et al., 2022). Thanks to the latest evidence, different strategies have been used to genetically modify *Komagataeibacter* strains (Florea et al., 2016). Mainly, bacterial strains have been engineered via the knockdown of target genes, gene overexpression or the expression of foreign genes. Gene disruption is a widely used technique for the study of cellulose production. An example is the inactivation of the glucose dehydrogenase (GDH) gene responsible for the synthesis of gluconic acid from glucose. The GDH mutant can use glucose to produce BC without producing gluconic acid as a by‐product, thus increasing substrate utilization efficiency for cellulose biosynthesis. Indeed, BC production from the GDH mutant was higher than that of the wild‐type strain by about 40% in static cultures and 230% in shaken conditions (Jacek et al., 2019; Kuo et al., 2015). High BC yield in *K. xylinus* could also be achieved through the partial or complete overexpression of the cellulose synthesis machinery from a heterologous plasmid bearing an extra copy of the *bcsABCD* operon. The resulting engineered *K. xylinus* strains produced BC faster and with higher yields (2‐ to 4‐fold) compared to the wild‐type strain (Jacek et al., 2019). Another example of genetic improvement is the introduction of foreign genes to generate implemented strains with new abilities, as is the case of the unprecedented use of lactose as a carbon source by *K. xylinus*. For this purpose, the promoter‐free *lacZ* gene encoding β‐galactosidase, one of the lactose operon genes, was inserted into the chromosomal DNA of *K. xylinus* by random transposon mutagenesis, generating a lactose‐utilizing and cellulose‐producing mutant strain (Battad‐Bernardo et al., 2004).

BC has the same molecular formula as plant cellulose but presents several distinct advantages compared with plant cellulose, making it an excellent candidate for several applications. BC is pure cellulose free of lignin, hemicellulose and pectin, so no extra processing is required for polymer purification. BC exhibits a sophisticated 3D porous network structure of cellulose nanofibres, with a diameter of 20–100 nm, thus providing high porosity and unique mechanical properties. Additionally, BC possesses other attractive physical properties, such as high crystallinity (70%–80%), high polymerization degree, high water content of up to 99% and moldability (Hu et al., 2014). For all these characteristics, BC is a versatile material exploitable for many applications (individually or in combination with different components) in the commercial areas of biomedicine, textile, food industry, personal care products, household chemicals, composite materials, etc.

Biomedicine is one of the most explored fields in BC usage. Indeed, BC is a natural nanomaterial and, compared with other synthetic polymers, presents certain advantages such as biocompatibility, non‐toxicity, biodegradability and non‐carcinogenic activity. Currently, the main commercial utilization of BC membranes is as wound‐dressing devices (Picheth et al., 2017). The molecular structure of BC confers some intrinsic features required for wound dressing, especially the high water‐holding capacity (WHC) and the water‐release rate (WRR). BC dressings are claimed to have the ability to simultaneously maintain moisture and absorb exudates from the injured tissue, thus avoiding infections, reducing local pain and accelerating skin recovery. These properties may be controlled through the structural modification of BC with specific additives, which confer a different available pore volume, and consequently an altered WHC/WRR ratio (Sulaeva et al., 2015). As an example, chitosan alginate and other highly hydrophilic compounds are used to obtain a pore size reduction of BC, increasing the WHC/WRR parameters. As a result, BC membranes reinforced with both chitosan and alginate present higher elongation, rehydration, swelling ratios and water vapour transmission (Chang & Chen, 2016).

Although BC possesses unique intrinsic mechanical and chemical properties, numerous modification methods have been investigated to open up possibilities for improving BC with new functionalities. In this context, composite generation is an approach to provide new properties to the existing BC‐based materials required for a specific application. One of the major complications in the wound‐healing process involves contamination with pathogenic organisms that cause infections and inflammation, hence physical and biological BC optimizations have been developed to overcome these problems (Portela et al., 2019; Swingler et al., 2021). Active compounds can be loaded to BC membranes mainly by (i) immersion/saturation, (ii) by chemical modification, since BC presents a large number of exposed hydroxyl groups that can be functionalized, and (iii) by genetic engineering. The most common and simplest method of BC complementation is via immersion in the compound solution, although the process is time‐consuming (Swingler et al., 2021). The choice of the implementation methods depends on the BC type and mostly on the properties of the active compound (i.e. molecular size, solubility, stability and working concentration). Impregnation has been used to load anti‐microbial agents such as polyhexanide (PHMB), octenidine and photoactive TiO_2_ or to develop topical BC drug delivery systems. BC wound‐dressing devices can act as drug reservoirs in many clinical applications, such as superficial skin infections, burns or cancer lesions. Many drugs such as tetracycline, diclofenac, ibuprofen or lidocaine have been successfully implanted into the BC structure and applied as transdermal or local delivery systems, avoiding adverse gastro‐intestinal symptoms (Picheth et al., 2017; Portela et al., 2019; Sulaeva et al., 2015; Swingler et al., 2021). Silver nanoparticle BC membranes are among the most important composites already described and utilized as wound dressings for their bacteriostatic and bactericide properties. These composites present the great advantage of not generating antibiotic resistance, an emergent global health problem, as they are non‐toxic and show good biocompatibility (Picheth et al., 2017; Sulaeva et al., 2015). Furthermore, silver BC materials are transparent, allowing a continuous clinical observation of the healing progress (Portela et al., 2019). One of the latest, modern strategies to accelerate the wound healing process is the incorporation of human cells into BC membranes or BC hydrogel. Recent studies have demonstrated the high regenerative potential of BC associated with mesenchymal stem cells or epidermal keratinocytes and dermal fibroblasts (Loh et al., 2018).

BC has also been commercially exploited as a source of raw material for the textile and shoe industry. As mentioned above, BC is a remarkable material; it is malleable, over 10 times stronger than plant cellulose, and biocompatible. Despite its unique properties, some BC features can be improved still further to obtain a flexible, breathable and water‐impermeable material. Fernandes et al. (2019) produced a BC nanocomposite through the exhaustion process to incorporate two hydrophobic polymers into the cellulosic porous network. The generated BC composite showed an increased nanofibre thickness, a higher hydrophobicity and a reduced water vapour permeability, while maintaining an adequate level of breathability (Fernandes et al., 2019). The mechanical properties of the new composite material make it a potential candidate to be used in the fashion industry. Many companies and researchers are betting on BC as a new eco‐friendly material that could partially substitute leather (García & Prieto, 2019). Recently, the Modern Synthesis Company introduced a bio‐fabrication process to produce a new class of material that uses bacteria to weave customizable bio‐textiles and composites. The company created shoes from *K. rhaeticus*, and is also developing a multigene system to add strength, stability and greater speed to the beta sheets they manufacture (Melton, 2022). Furthermore, BC‐hydrogel with optimized rheological properties is already being used as ink in 3D printing for advanced functional applications in medical, electronic and smart textile biomaterials (García & Prieto, 2019; Swingler et al., 2021).

To sum up, BC is a unique functional material with a high level of chemical purity, crystallinity and a nano‐fibrillar matrix. Moreover, it lends itself to modifications and composite generation to improve its properties and make it suitable for multiple applications. BC is, therefore, a highly adaptable material that could provide a viable alternative to combat the use of petroleum‐based analogues, and could play a vital role in the transition to the new circular economy (Swingler et al., 2021). However, the cost of BC‐based products is apparently higher than that of plant‐derived cellulose. Typically, the cost of BC production accounts for around 30% of the total product, limiting the large‐scale production and commercialization of BC products (Manan et al., 2022; Zhong, 2020). The high production cost is mainly due to the lack of an efficient fermentation system; therefore, more attempts should be made to improve the industrial process and the yield of BC. The scientific community and the industrial sector should unite their efforts to achieve more efficient operations and obtain novel and efficient BC‐producing microbial strains through genetic engineering.

## AUTHOR CONTRIBUTIONS

**Sophie‐Marie Martirani‐VonAbercron:** Conceptualization (equal); writing – original draft (equal); writing – review and editing (equal). **Daniel Pacheco‐Sánchez:** Conceptualization (equal); writing – original draft (equal); writing – review and editing (equal).

## CONFLICT OF INTEREST STATEMENT

The authors declare no conflict of interest.
